# Associations between school backpack loading, sitting time and low back pain among schoolchildren in Gaborone, Botswana

**DOI:** 10.4102/hsag.v31i0.3415

**Published:** 2026-05-26

**Authors:** Ngonidzaishe B. Mudiwa, Mariette Swanepoel, Terry J. Ellapen

**Affiliations:** 1 Department of Physical Activity, Sport and Recreation, Faculty of Health Sciences, North-West University, Potchefstroom, South Africa; 2 Department of Sport, Rehabilitation and Dental Sciences, Faculty of Science, Tshwane University of Technology, Pretoria, South Africa

**Keywords:** low back pain, school backpack load, sitting time, ergonomics, schoolchildren, Botswana

## Abstract

**Background:**

Low back pain (LBP) is increasingly reported among school-aged children, a population vulnerable to musculoskeletal strain as a result of growth-related changes, developing spinal structures and school demands such as heavy backpacks and prolonged sitting. Although international evidence highlights these risk factors, data from African contexts, particularly Botswana, remain limited.

**Aim:**

This study investigated associations between school backpack loading, sitting time and the prevalence and intensity of LBP among 10–12-year-old schoolchildren.

**Setting:**

Primary schools in Gaborone, Botswana.

**Methods:**

A cross-sectional study was conducted among 521 children (46% boys; mean age = 11.1 ± 0.7 years). Backpack and body mass were measured using calibrated scales, and sitting time was self-reported using the International Physical Activity Questionnaire – Short Form (IPAQ-SF). Low back pain frequency and intensity were assessed using the Young Spine Questionnaire (YSQ). Chi-square tests examined associations between LBP outcomes and independent variables, and Cramér’s V interpreted effect sizes.

**Results:**

Approximately 43% carried backpacks exceeding 10% of body mass; 34% reported frequent LBP and 14% moderate-to-severe intensity. Heavier loads were weakly but significantly associated with more frequent LBP (χ^2^ = 4.05; *p* = 0.044; Φ = 0.09). No associations were found with sitting time, age or sex.

**Conclusion:**

Heavier backpack loads were associated with higher LBP frequency among Gaborone schoolchildren, although the effect size was small. These findings highlight the importance of addressing modifiable ergonomic factors, particularly load management, in school health strategies.

**Contribution:**

This study highlights modifiable ergonomic risk factors for LBP in Botswana and supports the inclusion of load management and posture education in school health programmes.

## Introduction

Low back pain (LBP) has emerged as a significant musculoskeletal health concern among school-aged children worldwide, with prevalence estimates ranging from approximately 25% – 55% and evidence indicating a growing burden in this population (Ambrosio et al. 2023; Calvo-Muñoz, Gómez-Conesa & Sánchez-Meca 2013; Fazaa et al. 2024; Wirth, Knecht & Humphreys 2013). Once regarded as an adult condition, LBP is now increasingly reported during childhood and adolescence, with prevalence estimates ranging from approximately 25% – 55% in this population (Calvo-Muñoz et al. 2013; Wirth et al. 2013; Noll et al. 2016; Masiero et al. 2021). More recent evidence suggests that this burden may be increasing, driven by behavioural, lifestyle and school-related ergonomic factors (Ambrosio et al. 2023). Early onset of LBP is particularly concerning, as symptoms during childhood are associated with a higher likelihood of persistence into adulthood, contributing to chronic musculoskeletal disorders, functional limitations and reduced quality of life (Hartvigsen et al. 2018; Kovacs et al. 2003; Minghelli 2017). One of the most commonly reported modifiable risk factors for LBP in children is school backpack loading. International recommendations generally suggest that backpack mass should not exceed 10% – 15% of a child’s body weight; however, studies consistently report that many children exceed these thresholds (Akbar et al. 2019; Brackley & Stevenson 2004; Dianat et al. 2013; Ibrahim 2012; Lopez Hernandez et al. 2020). Increasing academic demands, additional learning materials and limited access to storage facilities have contributed to progressively heavier schoolbags in many settings. Beyond total load, the manner in which backpacks are carried may also influence spinal loading. Carrying a backpack asymmetrically, such as over one shoulder, may result in lateral spinal deviation, while excessive loads increase compressive forces on the lumbar spine and require compensatory postural adaptations, including forward trunk inclination and altered gait patterns (Cuenca-Martínez et al. 2023; Dockrell, Simms & Blake 2015). Experimental and observational evidence indicates that increasing backpack loads negatively affect gait parameters, balance and plantar pressure distribution, particularly at loads exceeding 10% – 15% of body weight (Bukowska et al. 2021; Tomal et al. 2022). These biomechanical changes have been associated with an increased likelihood of musculoskeletal discomfort and pain among schoolchildren (Grobler & Kramer 2023). However, evidence regarding the relationship between backpack load and LBP remains inconsistent, suggesting that this association is complex and likely influenced by additional factors such as posture, physical activity and individual susceptibility (Adeyemi, Rohani & Rani 2017; Sankaran et al. 2021). Recent systematic and biomechanical evidence further supports the view that LBP in children is multifactorial, with no single exposure acting as an independent determinant (Bukowska et al. 2021; Ji et al. 2024; Jenčíková, Kasović & Zvonař 2024). Beyond backpack carriage, sedentary behaviour is increasingly recognised as a contributor to musculoskeletal discomfort in children. School-aged learners spend a substantial proportion of their waking hours sitting, often in prolonged and uninterrupted bouts during classroom activities, homework and screen-based leisure (Arundell et al. 2019; Chaput et al. 2020). Prolonged sitting and static postures may increase spinal loading, reduce trunk muscle activation and contribute to postural imbalance. These effects may be exacerbated by poorly designed classroom furniture that does not adequately accommodate children’s anthropometric characteristics, leading to sustained suboptimal sitting postures (Acar, Erdil & Ozcan 2023; Loredan et al. 2022). Recent evidence suggests that higher levels of sedentary behaviour are associated with an increased likelihood of musculoskeletal pain, including low back and neck pain in adolescents (Da Costa et al. 2022; Mahdavi et al. 2022). However, the strength and nature of this relationship remain unclear. A recent systematic review and meta-analysis reported only small associations between sedentary behaviour and spinal pain, with limited evidence supporting a causal relationship (Montgomery et al. 2024). These findings suggest that factors such as posture quality, physical activity levels and individual characteristics may mediate the relationship between sedentary behaviour and LBP. Despite growing global attention to LBP in children, limited research has focused on African settings. Available evidence indicates that musculoskeletal pain, including LBP, is relatively common among school-aged children in several African countries. For example, studies in Tunisia and South Africa have reported notable prevalence rates of spinal pain and demonstrated associations between schoolbag load, posture and musculoskeletal discomfort (Mwaka et al. 2014; Fazaa et al. 2024; Grobler & Kramer 2023). However, contextual differences in school environments, infrastructure and lifestyle behaviours limit the generalisability of these findings across regions. In Botswana, data on the combined influence of backpack loading and sedentary behaviour on LBP in children remain scarce. This highlights the need for context-specific research to inform targeted school health interventions and preventative strategies. Therefore, the aim of this study was to examine the associations between school backpack loading, sitting time and both the prevalence and intensity of LBP among 10–12-year-old schoolchildren in Gaborone, Botswana. It was hypothesised that children carrying backpacks weighing ≥ 10% of their body mass and those reporting longer sitting durations would report more frequent episodes of LBP and greater pain intensity.

## Research methods and design

### Study design

A quantitative, cross-sectional research design was employed to investigate the associations between school backpack loading, sitting time and LBP among schoolchildren. The design was suitable for identifying prevalence and relationships between categorical health-related variables in a natural school setting.

### Setting and participants

The study was conducted between August and September 2022 among primary schools in Gaborone, Botswana. Schools were randomly selected from five geographical clusters to ensure diverse socioeconomic representation. The target population consisted of children aged 10–12 years attending grades 5 and 6. A total of 521 participants (46% boys, 54% girls; mean age = 11.14 ± 0.74 years) were included in the final analysis. The initial estimated sample size of 543 was based on a 95% confidence level, a 5% margin of error and an anticipated response rate of 80%, ensuring adequate statistical power. Inclusion criteria required participants to be apparently healthy (i.e. children without known medical conditions or musculoskeletal disorders that could affect normal school participation, based on school records and self-report), to use school backpacks daily and to have obtained written parental consent and child assent. Children with physical disabilities or known spinal deformities were excluded based on available school records and participant and/or teacher reporting, as no clinical screening was conducted.

### Instrumentation

Three validated instruments were employed:

1. *Young Spine Questionnaire (YSQ)* – used to assess LBP frequency and intensity among children aged 9–12 years. The YSQ has demonstrated good test–retest reliability (intraclass correlation coefficient [ICC]: 0.69–0.79) (Lauridsen & Hestbaek 2013). Low back pain frequency was categorised as *never, rarely, sometimes* or *often*, while intensity was rated from *none* to *very severe pain*. For analysis purposes, LBP outcomes were operationally defined using dichotomised categories: Frequency was classified as ‘no or infrequent pain’ (*never/rarely*) and ‘frequent pain’ (*sometimes/often*), while intensity was categorised as ‘no-to-mild pain’ (*none, very mild, mild*) and ‘moderate-to-severe pain’ (*moderate, severe, very severe*).
2. *International Physical Activity Questionnaire – Short Form* (*IPAQ-SF*) – applied to estimate weekday sitting time (ICC: 0.64–0.74). Sitting time reflected self-reported total sitting duration on a typical weekday, including time spent sitting at school, during homework and in leisure activities. Sitting time was dichotomised as < 8 h/day or ≥ 8 h/day, based on thresholds used in previous paediatric sedentary behaviour studies (Ridgers et al. 2015).
3. Anthropometric and Backpack Measures – Body mass and backpack mass were measured using a calibrated digital scale to the nearest 0.1 kg, with participants wearing light school clothing and empty pockets. Backpack load was expressed as a percentage of body mass and classified as *< 10% body weight (normal)* or ≥ *10% body weight (heavy)* (Puckree, Silal & Lin 2004; Spiteri et al. 2017). Body mass index (BMI) was calculated as kg/m^2^ and interpreted using age- and sex-specific Fitnessgram Healthy Fitness Zone (HFZ) standards, rather than adult classifications, to ensure appropriate paediatric interpretation. Percentage body fat (%BF) was estimated from triceps and calf skinfold thickness measurements using standard procedures and sex-specific prediction equations as outlined in the Fitnessgram protocol. Measurements were obtained using a calibrated skinfold calliper, and %BF values were classified according to Fitnessgram categories: HFZ, Needs Improvement (NI) and Needs Improvement – Health Risk (NI-HR).

### Procedure

Following institutional and governmental permissions, the researcher visited each selected school to introduce the study. The purpose and procedures were explained to teachers, parents and participants to ensure comprehension and voluntary participation. Data collection was conducted in classrooms by the primary researcher, assisted by trained research assistants, under standardised conditions. Research assistants were trained prior to data collection to ensure consistency in questionnaire administration and measurement procedures. Participants completed the YSQ and IPAQ-SF individually, with trained research assistants available to clarify items. Anthropometric and backpack measurements were recorded immediately afterwards. The entire process, including briefing and completion, took approximately 45 min/class.

### Statistical analysis

Data were analysed using the Statistical Package for the Social Sciences (SPSS) version 29.0 (IBM, Armonk, NY, United States). Descriptive statistics were used to summarise demographic characteristics and main study variables. Missing data were present primarily for the sitting time variable and, to a lesser extent, for LBP variables. Analyses were conducted on valid cases only, using listwise deletion for the relevant cross-tabulations. Accordingly, the denominators for LBP frequency, LBP intensity and sitting time differ across analyses, and these valid sample sizes are reported in the Results section and accompanying tables. To meet the assumptions for cross-tabulation, LBP frequency was recoded into two categories: *No or infrequent pain* (combining ‘never’ and ‘rarely’) and *frequent pain* (combining ‘sometimes’ and ‘often’). Low back pain intensity was similarly categorised as *no-to-mild pain* (none, very mild, mild) and *moderate-to-severe pain* (moderate, severe, very severe). Chi-square (χ^2^) tests were used to assess associations between LBP outcomes and categorical predictors (age, sex, backpack mass and sitting time). Assumptions for chi-square analyses, including expected cell counts, were evaluated prior to analysis, and category collapsing was applied where necessary. Effect sizes were interpreted using Phi (Φ) or Cramér’s V, with the following thresholds: 0–0.10 = very weak, 0.10–0.30 = weak, 0.30–0.50 = moderate, 0.50–0.70 = strong, ≥ 0.70 = very strong association. Statistical significance was accepted at *p* < 0.05.

### Ethical considerations

Ethical clearance was granted by the Health Research Ethics Committee (HREC) of the North-West University (reference: NWU-00185-21-A1) and approved by the Botswana Ministry of Education and participating schools. Written parental consent and child assent were obtained prior to the participant’s involvement. Confidentiality and anonymity were maintained throughout the study, and participants could withdraw at any time without consequence.

## Results

The demographic, anthropometric, backpack-related and sitting-time characteristics of the study cohort, including the distribution of LBP frequency and intensity are presented in Table 1.

**TABLE 1:** Demographic, anthropometric, backpack-related and sitting-time characteristics of the study cohort, including the distribution of low back pain frequency and intensity.

Variables	Percentiles			*n*	Valid per cent (%)	Categorisation for LBP frequency and LBP intensity (%)	Missing values	
	25	50	75				*n*	%
**Continuous variables**								
**Age (years) *n* = 521**								
Mean ± s.d.: 11.14 ± 0.74	11.00	11.00	12.00	-	-	-	-	-
Mean ± s.d.: 19.30 ± 3.82	16.25	18.85	21.63	-	-	-	-	-
**Body mass (kg) *n* = 521**								
Mean ± s.d.: 42.26 ± 9.58	36.00	41.00	46.35	-	-	-	-	-
**Backpack mass (kg) *n* = 521**								
Mean ± s.d.: 4.04 ± 1.32	3.00	4.00	4.85	-	-	-	-	-
**Backpack mass as a % of body mass (%) *n* = 521**								
Mean ± s.d.: 9.95 ± 3.78	7.14	9.23	11.90	-	-	-	-	-
**Sitting time on a weekday (h/day) *n* = 521**								
Mean ± s.d.: 7.39 ± 2.43	6.00	7.00	9.00	-	-	-	-	-
**Categorical variables**								
Age classification	-	-	-	521	100	-	-	-
10-year-olds	-	-	-	110	21	-	0	0
11-year-olds	-	-	-	229	46	-	-	-
12-year-olds	-	-	-	182	35	-	-	-
Sex classification	-	-	-	521	100	-	-	-
Boys	-	-	-	240	46	-	0	0
Girls	-	-	-	281	54	-	-	-
Frequency of LBP	-	-	-	484	93	-	37	7
Never^#^	-	-	-	199	38	59	-	-
Rarely^#^	-	-	-	107	21	-	-	-
Sometimes*	-	-	-	134	26	34	-	-
Often*	-	-	-	44	8	-	-	-
Intensity of LBP	-	-	-	484	93	-	37	7
None^##^	-	-	-	200	38	78	-	-
Very mild pain^##^	-	-	-	135	26	-	-	-
Mild pain^##^	-	-	-	73	14	-	-	-
Moderate pain**	-	-	-	45	9	14	-	-
Severe pain**	-	-	-	18	3	-	-	-
Very severe pain**	-	-	-	13	2	-	-	-
Backpack mass classification	-	-	-	521	100	-	0	0
< 10% of body weight	-	-	-	296	57	-	-	-
≥ 10% of body weight	-	-	-	225	43	-	-	-
Sitting time classification	-	-	-	200	38	-	321	62
< 8 h/day	-	-	-	121	23	-	-	-
≥ 8 h/day	-	-	-	79	15	-	-	-

Note: Values are presented as mean ± s.d. for continuous variables and *n* (%) for categorical variables. Backpack load is expressed as a percentage of body mass, and sitting time was measured using the IPAQ-SF. Low back pain frequency and intensity were derived from the Young Spine Questionnaire (YSQ). To satisfy crosstabulation test assumptions, LBP frequency categories were collapsed as follows: ‘Never’ (#) and ‘Rarely’ (#) were combined into *No or infrequent LBP*, and ‘Sometimes’ (*) and ‘Often’ (*) were combined into *Frequent LBP*. Concerning LBP intensity, ‘None’ (##) and ‘Very mild’ (##) were recoded as *No-to-mild LBP intensity*, ‘Moderate’ (**), ‘Severe’ (**) and ‘Very severe’ (**) were combined to *Moderate-to-severe LBP intensity*.

LBP, low back pain.

### Participant characteristics

A total of 521 schoolchildren (mean age: 11.14 ± 0.74 years) participated in the study. The sample comprised 46% boys (*n* = 240) and 54% girls (*n* = 281). The mean body mass was 42.26 kg ± 9.58 kg, and the mean backpack mass was 4.04 kg ± 1.32 kg, corresponding to 9.95 ± 3.78% of body mass. Based on classification criteria, 43% of participants (*n* = 225) carried backpacks weighing ≥ 10% of their body mass.

### Sitting time (explicit missing data handling)

Self-reported weekday sitting time averaged 7.39 ± 2.43 h/day. However, sitting time data were available for only 200 participants (38%), with 321 participants (62%) having missing data. Among those with valid responses, 60% (*n* = 121) reported sitting for less than 8 h/day, while 40% (*n* = 79) reported sitting for 8 h/day or more.

### Low back pain frequency

Low back pain frequency data were available for 484 participants (93%), with 37 (7%) missing responses. Among valid responses, 41% (*n* = 199) reported never experiencing LBP, 22% (*n* = 107) rarely, 28% (*n* = 134) sometimes and 9% (*n* = 44) often. When categorised for analysis, 63% of participants were classified as having no or infrequent LBP, while 37% were classified as having frequent LBP.

### Low back pain intensity

Low back pain intensity data were available for 484 participants (93%), with 7% missing. Among valid responses, 41% (*n* = 200) reported no pain, 28% (*n* = 135) very mild pain, 15% (*n* = 73) mild pain, 9% (*n* = 45) moderate pain, 4% (*n* = 18) severe pain and 3% (*n* = 13) very severe pain. For analytical purposes, 78% of participants were classified as experiencing no-to-mild pain, while 14% were classified as having moderate-to-severe pain.

### Associations between low back pain and study variables

Table 2 presents the associations between LBP frequency and selected categorical variables. A statistically significant association was observed between backpack mass classification and LBP frequency (χ^2^ = 4.052, *p* = 0.044), with a small effect size (Φ = 0.09). A higher proportion of frequent LBP was observed among children carrying backpacks weighing ≥ 10% of their body mass (12.6%) compared to those carrying lighter loads (7.2%).

**TABLE 2:** Associations between low back pain frequency and categorical variables (age, sex, backpack mass and sitting time).

Variable	No or infrequent LBP		Frequent LBP		χ^2^	*df*	*p*-value	Phi	Cramér’s V
	%	*n*	%	*n*					
**Age classification**									
10-year-olds	86.5	90	13.5	14	5.108	2	0.078	-	0.103
11-year-olds	89.4	195	10.6	23	-	-	-	-	-
12-year-olds	94.4	153	5.6	9	-	-	-	-	-
**Sex classification**									
Boys	91.0	201	9.0	20	0.098	1	0.755	0.014	-
Girls	90.1	237	9.9	26	-	-	-	-	-
**Backpack mass classification**									
< 10% body weight	92.8	258	7.2	20	4.052	1	0.044*	0.091	-
≥ 10% body weight	87.4	180	12.6	26	-	-	-	-	-
**Sitting time classification**									
< 8 h/day	92.9	104	7.1	8	2.163	1	0.141	0.108	-
≥ 8 h/day	86.3	63	13.7	10	-	-	-	-	-

χ^2^ = Chi-square; *df* = degrees of freedom; *p < 0.05. Phi/Cramer’s V Φ/V = 0: No association between the variables. 0 < Φ/V ≤ 0.10: Very weak association. 0.10 < Φ/V ≤ 0.30: Weak association. 0.30 < Φ/V ≤ 0.50: Moderate association. 0.50 < Φ/V ≤ 0.70: Strong association. Φ/V > 0.70: Robust association.

LBP, low back pain.

Table 3 presents the associations between LBP intensity and categorical variables. No statistically significant associations were observed between LBP intensity and age (χ^2^ = 2.865, *p* = 0.239; V = 0.08), sex (χ^2^ = 0.151, *p* = 0.698; Φ = 0.02), backpack mass classification (χ^2^ = 0.007, *p* = 0.934; Φ = 0.00) or sitting time classification (χ^2^ = 0.314, *p* = 0.575; Φ = 0.04).

**TABLE 3:** Associations between low back pain intensity and categorical variables (age, sex, backpack mass and sitting time).

Variable	No-to-mild pain intensity		Moderate-to- severe pain intensity		χ^2^	*df*	*p*-value	Phi	Cramér’s V
	%	*n*	%	*n*					
**Age categories**									
10-year-olds	62.5	65	37.5	39	2.865	2	0.239	-	0.077
11-year-olds	71.6	156	28.4	62	-	-		-	-
12-year-olds	70.4	114	29.6	48	-	-		-	-
**Sex classification**									
Boys	68.3	151	31.7	70	0.151	1	0.698	−0.018	-
Girls	70.0	184	30.0	79	-	-		-	-
**Backpack mass classification**									
< 10% body mass	69.1	192	30.9	86	0.007	1	0.934	−0.004	-
≥ 10% body mass	69.4	143	30.6	63	-	-		-	-
**Sitting time classification**									
< 8 h/day	69.1	77	30.9	35	0.314	1	0.575	−0.041	-
≥ 8 h/day	72.6	53	27.4	20	-	-		-	-

χ^2^ = Chi-square; *df* = degrees of freedom; *p < 0.05. Phi/Cramer’s V Φ/V = 0: No association between the variables. 0 < Φ/V ≤ 0.10: Negligible or very weak association. 0.10 < Φ/V ≤ 0.30: Weak association. 0.30 < Φ/V ≤ 0.50: Moderate association. 0.50 < Φ/V ≤ 0.70: Strong association. Φ/V > 0.70: Robust association.

LBP, low back pain.

### Summary of findings

The main finding of this study was that backpack mass was significantly, albeit weakly, associated with LBP frequency. Heavier backpacks were linked to more frequent LBP episodes among children aged 10–12 years. In contrast, no significant associations were found between sitting time, age or sex and either LBP frequency or intensity.

## Discussion

This study examined the associations between school backpack loading, sitting time and LBP among schoolchildren in Gaborone, Botswana. The main findings indicate that backpack load relative to body mass was associated with LBP outcomes although the strength of this relationship was modest. Sitting time, age and sex were not significantly associated with LBP, suggesting that factors beyond sedentary behaviour or demographic characteristics contribute to spinal discomfort. Overall, these findings indicate that LBP in children is multifactorial, involving interacting mechanical, behavioural and environmental factors (Ambrosio et al. 2023; Hartvigsen et al. 2018).

### Backpack loading and low back pain

The findings on backpack loading are consistent with the existing literature, indicating that excessive schoolbag weight may contribute to musculoskeletal discomfort in children. Previous studies have demonstrated that loads exceeding 10% – 15% of body weight are associated with alterations in posture, gait mechanics and spinal loading (Brackley & Stevenson 2004; Cuenca-Martínez et al. 2023). Experimental evidence further suggests that increased backpack loads result in forward trunk inclination, altered balance and increased plantar pressure, which may contribute to musculoskeletal strain (Bukowska et al. 2021; Tomal et al. 2022). In the present study, although backpack load was associated with LBP frequency, the relatively weak association suggests that load alone may not sufficiently explain the occurrence of pain. This aligns with previous research reporting inconsistent associations between backpack weight and LBP, suggesting that factors such as posture, duration of carriage, physical conditioning and individual susceptibility may moderate this relationship (Dianat et al. 2013; Sankaran et al. 2021).

### Sitting time and postural factors

Sedentary behaviour was also examined in relation to LBP; however, no significant associations were observed. This aligns with recent literature suggesting that prolonged sitting may contribute to musculoskeletal discomfort through increased spinal loading, reduced trunk muscle activation and sustained static postures (Da Costa et al. 2022; Mahdavi et al. 2022). However, the current findings align with previous systematic reviews, which indicate that the relationship between sedentary behaviour and spinal pain is generally weak and inconsistent (Montgomery et al. 2024). This suggests that sitting duration alone is unlikely to be an independent determinant of LBP but may interact with factors such as posture quality, physical activity levels and ergonomic conditions. Together, these findings reinforce the idea that single behavioural exposures may not independently explain LBP risk in children.

### Contextual interpretation within the school environment

Importantly, these findings should be interpreted within the specific school context of Botswana, where environmental and infrastructural factors may influence both backpack use and sedentary behaviour. Limited access to storage facilities, the need to transport multiple textbooks daily and prolonged classroom-based learning may contribute to sustained mechanical loading of the spine. In addition, variability in classroom ergonomics and seating design may further exacerbate postural strain during extended periods of sitting. These contextual factors may interact with individual biomechanical and behavioural characteristics, potentially explaining the weak but significant association observed between backpack load and LBP frequency. This highlights the importance of considering both environmental and individual-level determinants when addressing musculoskeletal health in school-aged populations.

### Age and sex differences

No significant associations were observed for age or sex, suggesting that biomechanical and behavioural factors may be more relevant determinants of LBP at this stage of development.

### Strengths and limitations

This study has several strengths, including the concurrent assessment of both mechanical (backpack load) and behavioural (sitting time) factors associated with LBP in a previously under-researched population. The relatively large sample size and objective measurement of backpack and body mass further strengthen the robustness of the findings. However, certain limitations should be acknowledged. The cross-sectional design limits causal inference, and reliance on self-reported measures may introduce recall and reporting bias. In addition, sitting time data were available only for a subset of participants, which may limit the generalisability of findings on sedentary behaviour. Unmeasured factors such as psychosocial influences, physical fitness and growth-related changes may also contribute to LBP and were not accounted for in the present study. Although the YSQ and IPAQ-SF are validated instruments, no formal cultural adaptation or validation was conducted within the Botswana context, which may have influenced comprehension, response accuracy and the validity of self-reported data. Future research should consider longitudinal designs and objective measures of sedentary behaviour to better understand causal pathways.

### Practical implications

From a practical perspective, these findings suggest that interventions to reduce LBP among schoolchildren should adopt a multifactorial approach. Strategies may include promoting appropriate backpack loading practices; encouraging regular movement and breaks from prolonged sitting; participating in physical activity, exercise or active play during breaks and improving classroom ergonomics. Educational initiatives targeting both learners and educators may be beneficial in promoting healthy posture and movement behaviours within the school environment.

## Conclusion

Heavier backpack loads were significantly associated with more frequent LBP among schoolchildren aged 10–12 years in Gaborone. Although the effect size was modest, repeated exposure to excessive load may contribute to cumulative musculoskeletal strain. These findings reinforce the conclusion that LBP in children is multifactorial, involving interacting mechanical, behavioural and environmental determinants.
